# KALK study: ultrasound guided needling and lavage (barbotage) with steroid injection versus sham barbotage with and without steroid injection - protocol for a randomized, double-blinded, controlled, multicenter study

**DOI:** 10.1186/s12891-017-1501-9

**Published:** 2017-04-04

**Authors:** Stefan Moosmayer, Ole Marius Ekeberg, Hanna Bjørnsson Hallgren, Ingar Heier, Synnøve Kvalheim, Jesper Blomquist, Are Hugo Pripp, Nils Gunnar Juel, Stein Harald Kjellevold, Jens Ivar Brox, KALK study group, Unni S. Seljom, Anne V. Gärtner, Nina J. Kise, Line K. Løvereide, Dalia Martinkiene, Ida Dånmark

**Affiliations:** 1 Orthopedic Department, Martina Hansens Hospital, Dønskiveien 8, 1346 Gjettum, Norway; 2 Department for Physical Medicine and Rehabilitation, Helse Fonna Hospital, Stord, Tysevegen 64, Stord Sjukehus HF, 5416 Stord, Norway; 3 Orthopedic Department, Linköping University Hospital, Garnisonsvägen 10, 581 85 Linköping, Sweden; 4 Department for Physical Medicine and Rehabilitation, Vestfold Hospital, Stavern, Kysthospitalveien 61, 3294 Stavern, Norway; 5 Department for Physical Medicine and Rehabilitation, Oslo University Hospital, P.O.B. 4956, Nydalen, 0424 Oslo Norway; 6 Orthopedic Department, Haraldsplass Deaconess Hospital, P.O.B. 6165, 5892 Bergen, Norway; 7 Oslo Centre of Biostatistics and Epidemiology, Research Support Services, Oslo University Hospital, 0424 Oslo, Norway; 8 Department of Radiology, Haraldsplass Deaconess Hospital, P.O.B. 6165, 5892 Bergen, Norway

**Keywords:** Calcific tendinitis, Barbotage, Ultrasound guided lavage, Corticosteroids, Sham treatment, Placebo

## Abstract

**Background:**

For the treatment of calcific tendinitis of the shoulder a variety of treatment regimes exist. Commonly used treatment measures include medication with oral analgesics, corticosteroid injections, extracorporeal shockwave therapy, ultrasound guided needling and lavage, and surgical treatment. Earlier cohort studies suggest that patients may benefit from these treatments, but there are few randomized studies and conflicting evidence about the effectiveness of the various treatments. In the present study we aim to compare the effectiveness of ultrasound guided needling and lavage (barbotage) together with a steroid injection to sham barbotage with and without an additional steroid injection.

**Methods:**

The study will be performed in six secondary-care institutions in Norway and Sweden. It is designed as a pragmatic, randomized, three-arm, parallel group, double-blinded, sham-controlled clinical trial with a 2-year follow-up. It will be performed on 210 patients, aged 30 years or older, presenting with painful arc, positive impingement sign and a calcium deposit > 5 mm. Randomization to one of the three treatment options will be performed by using an online central randomization system. The three treatment groups are barbotage together with a subacromial steroid injection (the barbotage group), sham barbotage together with a subacromial steroid injection (the steroid group) or sham barbotage without a subacromial steroid injection (the placebo group). In the placebo group the steroid injection will be replaced by a short-acting local anaesthetic. Standardized home-based post-treatment physiotherapy will be performed by all patients for 8 weeks. Follow-ups are at 2 and 6 weeks, 4, 8, 12 and 24 months after treatment was given and will be performed with the patients and the outcome assessors blinded for group assignment. Primary outcome will be the Oxford shoulder score at 4 month follow-up. Secondary outcome measures are the QuickDASH upper extremity score, the EQ-5D-5L general health score and visual analogue scales for pain at rest, during activity, and at night.

**Discussion:**

The scientific evidence from this placebo-controlled trial will be of importance for future treatment recommendations in patients with calcific tendinitis.

**Trial registration:**

ClinicalTrials.gov: NCT02419040, registered 10 April 2015

EudraCT: 2015-002343-34, registered 23 September 2015 (retrospectively registered)

**Electronic supplementary material:**

The online version of this article (doi:10.1186/s12891-017-1501-9) contains supplementary material, which is available to authorized users.

## Background

Calcific tendinitis is a painful disorder of the shoulder of unknown etiology [1]. The condition is characterized by the formation of deposits of calcium crystals in one or several of the rotator cuff tendons. The prevalence of the condition has been reported to be 3 to 10% in the general population [2, 3] and 7 to 17% in individuals with shoulder pain [4]. Tendon inflammation located around the deposit is considered to contribute to pain. The course of the disease is often self-limiting with spontaneous calcium resorption and resolution of symptoms over several months [3]. In cases in which resorption is delayed, absent or incomplete, symptoms may persist and anti-inflammatory treatment and/or removal of the calcification may provide symptomatic relief. Different treatment methods including anti-inflammatory and analgesic medication, extracorporal shockwave therapy (ESWT), ultrasound guided needling and lavage (barbotage), and surgical treatment are in use, but a consensus on the preferred treatment is lacking.

Farin introduced barbotage as an ultrasound guided technique in 1995 [5]. It consists of needle aspiration and lavage of the calcium deposit. Both single and double needle procedures have been described [5–9]. Good short- and medium-term results have been reported from several cohort studies [6, 7, 10] . A systematic review of the efficacy of barbotage in the treatment of calcific tendinitis found the technique to be safe and effective with an estimated average of pain improvement of 55% [11]. Only few comparison studies between barbotage and other techniques have been performed. In one randomized study, barbotage was found to be superior to subacromial corticosteroid treatment [8]. Contrary, a systematic review and meta-analysis of minimally invasive therapies in the management of chronic calcific tendinopathy, concluded that barbotage was not more effective than subacromial corticosteroid injections. The review concluded that further research is needed to evaluate its effectiveness [12]. Considering the cyclic often self-limiting course of the disease, and the treatment’s anticipated placebo effect, the effectiveness of barbotage should preferably be answered by a sham-controlled randomized study.

### Aims

The aim of the present study is:

1. to compare the short-term effectiveness (at 4 months) of barbotage and steroid injection, sham barbotage and steroid injection, and sham barbotage without steroid injection in the treatment of calcific tendinitis, and
2. to compare the long-term effectiveness (2 years) between the study groups.

## Methods/Design

### Trial design

**Fig. 1:**
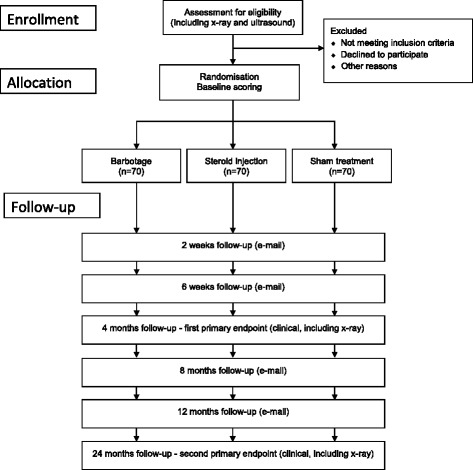
Patient flow through the study

The study will be performed at five hospitals in Norway and one hospital in Sweden as a pragmatic, randomized, three-arm, parallel group, double-blinded, sham-controlled superiority trial with a 2-year follow-up (Fig. 1). Departments of orthopaedics, radiology and physical medicine and rehabilitation will be involved in the conduction of the study. The recruiting sites are Martina Hansens Hospital, Sandvika; Helse Fonna Hospital, Stord; Haraldsplass Deaconess Hospital, Bergen; Vestfold Hospital, Stavern; Oslo University Hospital, Oslo; (all Norway), and Linköping University Hospital, Linköping, (Sweden). The population group that will be considered for study participation consists of patients referred from general practitioners from the catchment areas of the study hospitals. They represent the unselected group of patients with painful calcific tendinitis with inadequate effect from treatment measures performed in general practice care. Patients who will have to be excluded according to the criteria given in Tables 1 and 2 will be reported. Inclusion, treatment and follow-up of study patients will be performed by hospital based physicians, specialised in physical medicine or orthopaedic surgery, all with long experience and special interest in the treatment of shoulder patients. At one site the study intervention will be given by a radiologist. The study hospitals represent different types of hospitals ranging from university hospitals to municipal hospitals and from hospitals with an urban to those with a rural location. Study medication and equipment is available at all study sites as all study interventions are part of already existing treatment routines. The study will be conducted in compliance with the principles of the Declaration of Helsinki, the principles of Good Clinical Practice (GCP) and under consideration of national laws and regulations, and will be reported in accordance with the CONSORT guidelines.

**Table 1:** Inclusion criteria

Age of 30 years or older	
3 months or more of shoulder pain	
Moderate to severe pain localized on the top and/or lateral side of the shoulder, exaggerated by activities above shoulder level	
Pain at night when lying on the affected shoulder	
A painful arc [32]	
A positive Hawkin’s test [33] or Neer’s sign [34] for impingement	
A finding of one or more calcifications ≥5 mm in size on a standard anterior posterior radiograph, localized proximally to the greater tubercle, taken not more than 4 weeks prior to the intervention	
A sonographic finding of one or more calcifications ≥5 mm in size on the short or long axis view, localized in the supraspinatus or infraspinatus tendon	
A morphological radiographic appearance of Molé type A, B or C [13]	
The ability to understand written and spoken Norwegian (Swedish)	
An existing expected cooperation of the patient for the treatment and the follow-up

**Table 2:** Exclusion criteria

The presence of clinical and radiological signs of a recent spontaneous release of the calcific deposit such as a sudden change in size or density of the deposit on ultrasound together with an acute onset of extreme shoulder pain
Clinical signs of shoulder instability, glenohumeral arthritis, AC pathology, inflammatory arthropathy, fibromyalgia, frozen shoulder or cervical radiculopathy
Sonographic signs of a rotator cuff tear (full thickness or partial thickness) or of a tear or a dislocation of the long head of the biceps tendon
A history of surgery or barbotage of the relevant shoulder
A subacromial injection with a corticosteroid or treatment by ESWT during the last 3 months before inclusion
Medical contraindications for any of the invasive procedures
One of the following contraindications for the use of Lidocaine 10 mg/ml: Patients with serious hypovolaemia, known cardiac conduction disturbances, epilepsy or porphyrias, patients with known serious dysfunction of the liver or the kidneys
One of the following contraindications for the use of Triamcinolone 20 mg/ml: Patients with systemic infections unless specific anti-infective therapy is employed, patients with a local infection in the area of application, patients recently vaccinated with live vaccines, patients with known diabetes mellitus, renal or cardiac insufficiency, ulcerating colitis, gastric ulcer, psychosis, idiopathic thrombocytopenic purpura or ocular herpes simplex
Concomitant medication with one of the following medicinal products: Anti-arrythmics such as mexiletine or class III antiarrythmics (e.g. amiodarone), muscle relaxants (e.g. suxamethonium) or antipsychotics (e.g. pimozide, sertindole, olanzapine, quetiapine, zotepine, tropisetrone, dolasetron), antibiotics such as quinopristin/dalfopristin, anticoagulants suchas warfarin (if INR > 2) or novel oral anticoagulants
A history of prior allergic/hypersensitivity reactions related to the study medication
Knowledge of an ongoing pregnancy (fertile women not using contraception and who are uncertain whether they are pregnant or not will have to perform a pregnancy test
Nursing women

### Participants

Study participants will be recruited at the trial centers among patients referred from primary care services for treatment of a painful shoulder and in whom calcific tendinitis was diagnosed after clinical and radiological assessment. Eligibility for inclusion will be based on the presence of the inclusion criteria and the absence of the exclusion criteria given in Tables 1 and 2.

### Data collection at baseline

The following demographic and baseline data will be obtained at the primary consultation: Gender, age, shoulder affected (right, left, both), hand dominance, duration of symptoms, earlier treatment, use of analgesics, use of concomitant medication, comorbidities, occupational status, shoulder demanding activities at work or leisure time, and smoking habits. Standard shoulder radiographs (anterior-posterior, lateral and acromioclavicular views) will be obtained not more than 4 weeks prior to the intervention. The size and the type of the deposit will be recorded according to the Molé classification [13]. Diagnostic ultrasound of both shoulders will be performed and the size and the location of the deposits, related to the long head of the biceps tendon, will be recorded. On the treatment day, prior to treatment, three patient related outcome scores (Oxford Shoulder Score (OSS) [14, 15], quickDASH upper extremity score [16, 17], EQ-5D-5L general health score [18, 19]) and three visual analogue scales (VAS) for pain at rest, during activity and at night will be completed by the patient, together with the Stanford expectations of treatment scale (SETS) [20].

All study scores exist in a digital version in both languages. Patients fill in the scores directly on a computer/tablet through an internet website which is encrypted and has a secure protocol. Baseline and follow-up data will be stored on the research server at Martina Hansens Hospital, Sandvika, Norway. Access to the server is password protected and only the principal investigator (SM) has access to the database.

### Randomisation

Patients who fulfil the inclusion and exclusion criteria and who have signed the consent form after oral and written study information was given, will be randomized to one of the three treatment options. Randomization will take place on the day of the intervention by an online central randomization system (web-CRF) developed and administered by the Unit of Applied Cancer Research, Institute of Cancer Research and Molecular Medicine, Norwegian University of Science and Technology, Trondheim, Norway. To assure verifiability of the randomization, patient initials, year of birth and name of the treating hospital will have to be registered at the central randomisation database. After registration, the intervention group for the specific patient will be displayed on-screen. Allocation will be 1:1:1. Block randomization with varying block lengths and stratification according to hospital will be performed. The randomisation list will remain at the University of Trondheim for the whole duration of the study and, consequently, will be inaccessible for the investigators, care providers and outcome assessors at the study centres.

### Intervention

An orthopaedic surgeon (2 hospitals), a radiologist (1 hospital), or a specialist in physical medicine (3 hospitals), all with at least 5 years of experience in diagnostic and interventional ultrasound of the shoulder will conduct the allocated interventions. To ensure consistency a video of the procedure was produced and send to all trial sites (Additional file 1: Video barbotage procedure).

Interventions will be performed with the patient in supine position. The arm will be internally rotated to a degree that the treating physician considers to be the most favourable for the puncturing of the calcific deposit (usually arm on the back). An opaque sheet will be used to block the patient’s view of the screen of the ultrasound machine. After sterile skin preparation, with a 7 to 14 MHz transducer wrapped in a sterile drape, and by using sterile jelly, the calcific deposit will be sonographically identified, usually on the lateral transversal view. A 21-gauge needle will be introduced into the shoulder under sonographic guidance, and the pathway and the subacromial-subdeltoid bursa will be anesthetized with an injection of 10 ml of 1% lidocaine hydrochloride with adrenaline 5 μg/ml.

In patients randomized to barbotage a new 18-gauge needle connected to a 5 ml syringe with 4 ml of saline solution will be used to puncture the calcification using freehand technique under continuous sonographic monitoring. With the tip of the needle placed in the centre of the deposit, the calcification will be flushed. If backflow of calcific material can be identified in the syringe, lavage of the deposit will be performed by successive propulsion and aspiration with the syringe plunger. Extracted calcium can be visualized in the syringe as a cloudlike substance that settles at the bottom of the barrel. To avoid reinjection of the calcium into the deposit, the needle and the syringe will be hold in the horizontal plane during the procedure. The syringe will be substituted when the fluid has become cloudy and the procedure will be repeated until the backflow becomes clear. At the end of the procedure the content of the syringes will be poured into a measuring cup and, when settled at the bottom of the cup, the volume of the extracted calcium will be estimated. In cases where no material can be extracted, repeated perforation of the deposit will be performed to possibly initiate or accelerate spontaneous resorption.

In patients randomized to the steroid group or to the placebo group, the tip of the 18-gauge needle will be placed in the soft parts outside of the rotator cuff and movements mimicking the lavage procedure will be performed. A lavage procedure usually takes 5 min and a similar time period will be used for the mimicking manoeuvre.

Finally in all three groups, under sonographic monitoring, a new 21-gauge needle will be introduced into the subacromial bursa and 9 ml of Lidocaine hydrochloride 10 mg/ml and 1 ml of Triamcinolone 20 mg/ml (in the barbotage and the steroid group) or 10 ml of Lidocaine hydrochloride 10 mg/ml (in the placebo group) will be injected into the subacromial bursa.

### Blinding

The study will be conducted double-blinded with masking of patients and outcome assessors for treatment selection. We will blind the patient during the procedure by blocking their view of the ultrasound screen and the doctors working space and by mimicking a barbotage procedure in all patients. To control whether blinding of the patients was successful, patients will be asked after the treatment, and again after 2 and 6 weeks, which treatment they believe was performed. A blinding index as given in the literature will be calculated [21].

The specialists performing the study interventions will be excluded from all other tasks during the follow-up and the statistician conducting the statistical analysis will be blinded to the treatment given.

The specific treatment procedure will not be reported in the patient journal, only that the patient received the study treatment, according to randomisation. The specialist who performs the study treatment, will record the patient’s randomisation number, treatment selection, and name and date of birth in a decryption list which is kept in a safe place, accessible to the treating doctor only. This list can be consulted only if code break should be necessary during the trial. Code break is only permitted if knowledge of the study treatment seems mandatory for further treatment of a patient or if the patient insists in knowing the treatment group. All code breaks together with their causes will be recorded.

### Post-intervention treatment

In case of post interventional pain exacerbation non-prescription analgesics are recommended.

Routine use of the shoulder will be allowed without restrictions, but the patient is asked to avoid heavy shoulder labour for 2 weeks.

About 1 week after the intervention all patients will start on a standardized home-based physiotherapeutic treatment regime. The program consists of four shoulder exercises, and will be presented by written instructions, on photographic illustrations (Additional file 2: Folder physiotherapy procedure), and as a video (http://youtu.be/6nRYdqYniUI) [22]. Prior to the start of the program, a physiotherapist at each hospital will teach the patients how to perform the exercises correctly. All patients will have to maintain a regular protocol over 8 weeks during which they record each training session with date and number of exercises performed.

### Data collection at follow-up

Follow-up data will be collected 2 and 6 weeks, 4, 8, 12 and 24 months after the intervention (Table 1). At each time point the OSS [14, 15], the QuickDASH upper extremity score [16, 17], the EQ-5D-5L general health score [18, 19] and VAS for pain at rest, during activity, and at night will be filled in. To perform the 2 and 6 week and 8 and 12 month follow-ups patients will receive an e-mail with a link to a website, where they can fill in the study questionnaires at their computer at home.

At clinical follow-ups after 4 and 24 months study scores will be filled in on a computer/tablet at the hospital. The blinded assessor will register all treatment related adverse events by asking and by consulting each patient’s adverse event diary. The adverse event diary will be handed out to the patients on treatment day and will have to be kept over the entire 24-month follow-up period. Patients will be asked to enter in the diary any changes of their health condition that theiy perceive as an adverse event. The type of the event, its date of occurrence, its duration and its severity (on a 5 point Likert scale ranging from mild to severe) will have to be given. In case of a serious event, the hospital will have to be contacted immediately. If an adverse event requires treatment this will be recorded by the physician who is responsible for patient follow-up. The use and the dosage of prescription analgesics during the post treatment period will be recorded. An X-ray of the shoulder will be performed and, if still visible, the calcific deposit will be measured and classified according to Molé [13], blinded for baseline results.

### Outcome assessment

The study’s primary outcome measure is the OSS with the outcome at 4 months as the result of primary interest. Secondary outcomes are the results on the OSS at the other points of follow-up, results of the other study scores at all points of follow-up and the number of patients in each treatment group who change treatment during the study.

### Scoring instruments

All scoring instruments which will be used in the study are patient related outcome measures.

The OSS [14, 15] is a validated shoulder specific, 12-item score. It contains questions about pain and function and has been shown to be responsive to both surgical and non-surgical interventions on the shoulder. Its scoring method was modified in 2009 so that each of the 12 questions has five response categories scored from 4 (best) to 0 (worst) resulting in a total score ranging from 0 to 48 with a lower result indicating a greater degree of disability [15].

The QuickDASH upper extremity score [16, 17] is a shortened version of the DASH score with the number of items reduced from 30 to 11. It uses 5-point Likert scales to assess physical function and symptoms. It includes two domains for sport/art and work that are scored separately. The obtained value is transformed to a score ranging from 0 to 100 with a higher score indicating greater disability.

The Norwegian translation of the OSS and the Norwegian and Swedish version of the DASH score have been validated [23–25].

The EQ-5D-5L [18, 19] measures general health related quality of life. It comprises the dimensions mobility, self-care, usual activities, pain/discomfort, anxiety/depression each with 5 response categories, ranging from no problems to extreme problems. The score further includes a visual analogue scale for assessment of the health condition, ranging from the worst health (0) to the best health (100) you can imagine. Results can be presented as a health profile or as an index value.

Shoulder pain over the last week will be measured on three 0–10 visual analogue scales for pain at rest, during activities and at night. The scales are labelled no pain at the left end and worst imaginable pain at the right end.

The SETS [20] is a quick and easy-to-administer tool for the measurement of positive and negative pretreatment expectancies. The score consists of six items, three of them measuring positive expectancy, and three of them negative expectancy. It can be used to assess the influence of pretreatment expectancies on the outcomes in trials comparing real and sham treatment.

### Change of treatment

Patients who are still symptomatic or have redeveloped symptoms at four month follow-up or later, will be considered for supplementary treatment measures by the blinded follow-up assessor. If necessary, they will be offered treatment as usual, which means barbotage (repeated barbotage if barbotage was the primary treatment), steroid injection (repeated injection if injection was the primary treatment), ESWT, surgery, or therapist guided physiotherapy treatment, depending on findings and patient preferences. Earlier re-examination and change of treatment can be considered, in accordance with the Helsinki Declaration, for patients who are symptomatic to an extent that they cannot wait until the four month control. Patients who change the treatment during follow-up will remain in the study and will be followed-up according to an intention-to-treat principle. Unblinding of patients in conjunction with a change of treatment, will only be performed if the patient explicitly insists on knowing his primary treatment selection.

### Concomitant pain management

Previous treatment with analgesics is allowed but has to be stopped 48 h prior to baseline. In the post-treatment period the use of non-prescription analgesics such as Paracetamol (500 mg), Ibuprofen (600 mg) or Naproxen (250 mg) is permitted as it is considered part of the treatment. If post-treatment pain management necessitates the use of prescription analgesics the type and dosage will be recorded in the patients case report form at 4 and 24 month follow-ups.

### Harms

An adverse event diary will include all occurrences that the patient perceives as an adverse event. At follow-up, adverse events will be recorded by the follow-up assessor and will be sent to the sponsor. Once a year throughout the clinical trial, the sponsor will provide the respective Medicines Agency with an annual safety report. The format will comply with national requirements. If serious adverse events should occur, the sponsor will be informed immediately by phone or email.

### Sample size

Calculation of sample size was performed for an ANOVA of our primary outcome, which is the result on the OSS at 4 month follow-up. To detect a minimally important difference of 4 (SD 7) points [26] with a power of 90%, a 2-sided significance level of 0.05, 60 patients are required in each treatment group. To compensate for expected 15% drop-outs, we plan to include 70 patients in each treatment group. A supplementary analysis showed that this sample size also will be sufficient for pairwise post hoc *t*-test analyses (1 versus 2, 2 versus 3 and 1 versus 3), still with a significance level after Bonferroni correction of 0.017 but with a power of approximately 80%.

The actual statistical analysis of the interventions on primary outcome will be conducted using linear mixed models for repeated measurements adjusted for outcome measure at baseline. It is expected to give a slightly higher statistical power than an ANOVA.

### Statistical analysis

Demographic baseline data will be expressed for categorical variables as number of cases and for continuous variables by means with SD (if normally distributed) or by medians with range (if not normally distributed).

A linear mixed model for repeated measurements will be used for analysis. Because of an expected large number of crossovers after 4 months, the primary analysis will be performed on results up to 4 month follow-up and a secondary analysis on results up to 24 month follow-up. Analyses will be performed adjusted for baseline differences of the OSS and according to intention-to-treat. The linear mixed model will be estimated using linear maximum likelihood and include a random intercept, measure of the OSS at baseline as a covariate and observation time after intervention and type of intervention as factors. Mean differences (95% CI) between groups at 4 months follow-up will be presented from the linear mixed model to assess difference between interventions. A 2-sided *p*-value ≤ 0.05 is considered significant. Post-hoc pairwise comparisons for the primary outcome will be performed with p-value adjustments according to Bonferroni. Missing values will be handled by using mixed model analysis. Supplementary per-protocol analyses will be performed. A similar statistical analysis as described for the OSS will be used for the continuous secondary outcomes (QuickDASH, EQ-5D-5L).

Categorical variables will be expressed as numbers and percentages, and differences between groups will be analysed by the Chi^2^ or Fisher’s exact test. Possible associations between categorical baseline variables and outcomes will be explored by logistic regression analysis.

All subgroup analyses will be exploratory in nature and are planned for the size of the calcification at baseline (≤12.5 mm versus >12.5 mm), the volume of the removed material (≤0.1 ml versus > 0.1 ml), and the results on the Stanford expectation of treatment scale (positive expectancies versus negative expectancies). Selection of the threshold values for the size of the calcification at baseline and for the volume of the removed material are based on the findings of an unpublished pilot study from our institution.

Clinical safety will be investigated by assessing adverse events in a descriptive manner.

The statistician responsible for data analyses will blinded from the treatment allocation until completion of analyses.

## Discussion

Treatment of patients with long-standing symptoms from calcific tendinitis is controversial. If primary non-invasive treatment fails, different mini-invasive or non-invasive treatment options such as injection therapy, barbotage or ESWT exist. Which of them should be preferred is unclear.

Sham studies aiming to assess the effectiveness of invasive interventions for shoulder disorders are rarely performed but are necessary to provide a better understanding of the therapeutic mechanisms. In the present study we want to compare two mini-invasive approaches representing different therapeutic principles; in the steroid injection group symptomatic relieve will be tried to achieve by anti-inflammatory treatment alone and in the barbotage group by a supplementary removal of the calcific deposit. A literature search identified only one randomised study comparing these methods [8]. The study was performed on 48 patients and showed a significantly better result on the Constant score for the barbotage group at one year follow-up (86.0 versus 73.9 points). The present study will be based on a larger patient group and a longer follow-up, and will also include a placebo group. Inclusion of a placebo group is important as we aim to assess the contribution of the placebo response to treatment results. Comparison to placebo has traditionally been performed in drug studies. However, the existence of a surgical placebo effect can be assumed and may be underestimated [27]. This would apply in particular for the treatment of conditions where pain is the dominant symptom, as pain is the outcome most powerfully affected by placebo interventions [28]. Inclusion of a placebo group as a comparator in studies assessing the effectiveness of invasive or mini-invasive procedures has increased in recent years [29] and has revealed that the results of some surgical procedures are not different from placebo [30, 31]. Without a placebo group we cannot exclude that patients treated by cortisone injection or barbotage are exposed to an ineffective mini-invasive procedure with a profound placebo effect.

To ensure recruitment of a sufficient number of eligible patients, the study will be performed at six hospitals as a multicenter study. Based on the number of barbotage procedures performed at these hospitals in the time before study start, inclusion of ten patients per year and per site can be expected. To minimise the number of drop-outs, data capturing at four of the six follow-ups will be via email where the patients can fill in the study scores on a computer at home. Non-appearance of follow-up data will be noticed immediately on the central server and can be handled by sending out a reminder.

The aim of the present study is to contribute to better knowledge about the mechanisms for pain reduction and improvement of function in the treatment of patients with symptomatic calcific tendinitis.

## Additional files

Additional file 1: Video barbotage procedure. (AVI 256234 kb)

Additional file 2: Folder physiotherapy procedure. (DOCX 490 kb)

## Abbreviations

ESWT: Extracorporal shock wave therapy
GCP: Good Clinical Practice
OSS: Oxford shoulder score
SD: Standard Deviation
SETS: Stanford expectation of treatment score
VAS: Visual analogue scale
