# Supplementary material for: KALK study: ultrasound guided needling and lavage (barbotage) with steroid injection versus sham barbotage with and without steroid injection - protocol for a randomized, double-blinded, controlled, multicenter study
Source: BMC Musculoskelet Disord. 2017 Apr 4;18:138. doi: 10.1186/s12891-017-1501-9 (PMC5379547; doi:10.1186/s12891-017-1501-9)
Supplement: Supplementary file 2 — Folder physiotherapy procedure. (DOCX 490 kb) [file 12891_2017_1501_MOESM2_ESM.docx]

Instructions for how often the exercises should be performed and how many repetitions

Week 1 and 2: Exercise 1 and 2: Three times a day with 3 repetitions each.

Week 3 to 8: All exercises (1, 2, 3, 4): Once every day with 3 repetitions each.

**See video of the program at:** http://youtu.be/6nRYdqYniUI

**Diary**

Name:………………………………..

born: ………………………………..

| Day | Exercise 1 | | | Exercise 2 | | |
| --- | --- | --- | --- | --- | --- | --- |
| 1 |  |  |  |  |  |  |
| 2 |  |  |  |  |  |  |
| 3 |  |  |  |  |  |  |
| 4 |  |  |  |  |  |  |
| 5 |  |  |  |  |  |  |
| 6 |  |  |  |  |  |  |
| 7 |  |  |  |  |  |  |
| 8 |  |  |  |  |  |  |
| 9 |  |  |  |  |  |  |
| 10 |  |  |  |  |  |  |
| 11 |  |  |  |  |  |  |
| 12 |  |  |  |  |  |  |
| 13 |  |  |  |  |  |  |
| 14 |  |  |  |  |  |  |

| Day | Ex. 1 | Ex. 2 | Ex. 3 | Ex. 4 |
| --- | --- | --- | --- | --- |
| 28 |  |  |  |  |
| 29 |  |  |  |  |
| 30 |  |  |  |  |
| 31 |  |  |  |  |
| 32 |  |  |  |  |
| 33 |  |  |  |  |
| 34 |  |  |  |  |
| 35 |  |  |  |  |
| 36 |  |  |  |  |
| 37 |  |  |  |  |
| 38 |  |  |  |  |
| 39 |  |  |  |  |
| 40 |  |  |  |  |
| 41 |  |  |  |  |
| 42 |  |  |  |  |
| 43 |  |  |  |  |
| 44 |  |  |  |  |
| 45 |  |  |  |  |
| 46 |  |  |  |  |
| 47 |  |  |  |  |
| 48 |  |  |  |  |
| 49 |  |  |  |  |
| 50 |  |  |  |  |
| 51 |  |  |  |  |
| 52 |  |  |  |  |
| 53 |  |  |  |  |
| 54 |  |  |  |  |
| 55 |  |  |  |  |
| 56 |  |  |  |  |

| Day | Ex. 1 | Ex. 2 | Ex. 3 | Ex. 4 |
| --- | --- | --- | --- | --- |
| 15 |  |  |  |  |
| 16 |  |  |  |  |
| 17 |  |  |  |  |
| 18 |  |  |  |  |
| 19 |  |  |  |  |
| 20 |  |  |  |  |
| 21 |  |  |  |  |
| 22 |  |  |  |  |
| 23 |  |  |  |  |
| 24 |  |  |  |  |
| 25 |  |  |  |  |
| 26 |  |  |  |  |
| 27 |  |  |  |  |

**SHOULDER EXERCISES**

Home-based exercises for the shoulder

KALK study 2015

Sandvika · Oslo · Stavern · Stord · Bergen · Linköping

**
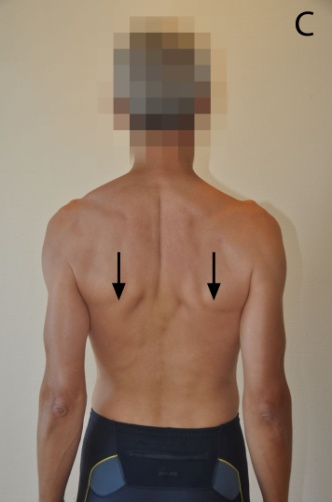

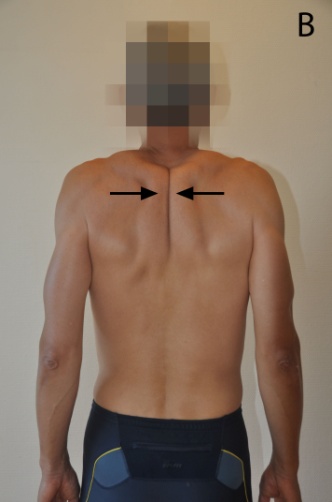

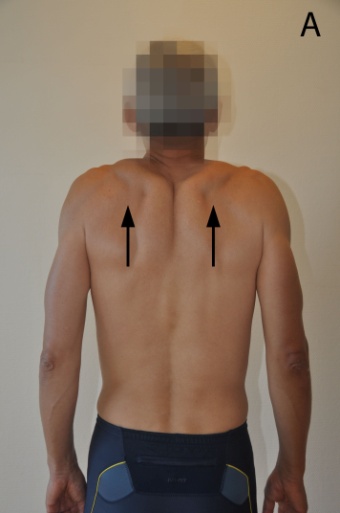
Exercise 1**

**Starting position:** Stand straight in front of a mirror. Arms hanging down by your side

**Exercise:** First shrug your shoulders (A), then squeeze your shoulder blades back and together (B), then pull them downwards as if putting them in your back pocket (C). Relax.

**Important:** Keep your arms relaxed during this exercise.

**
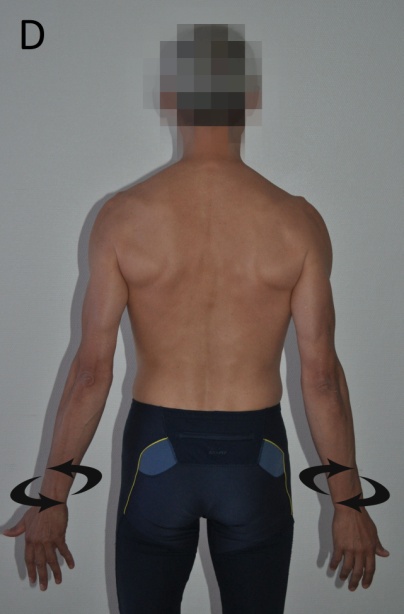

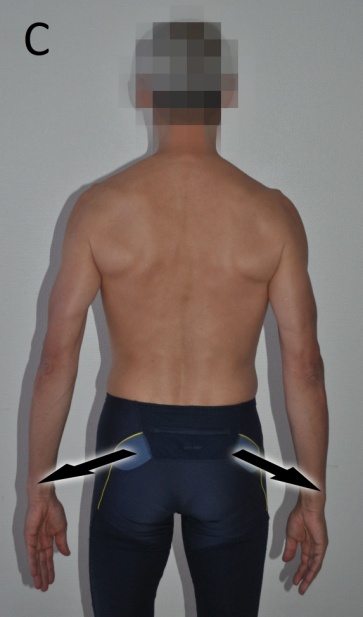

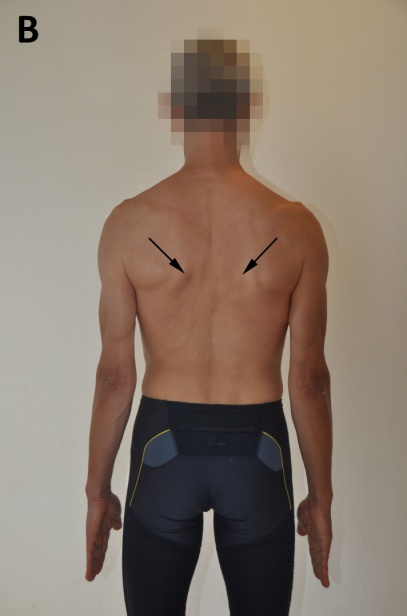

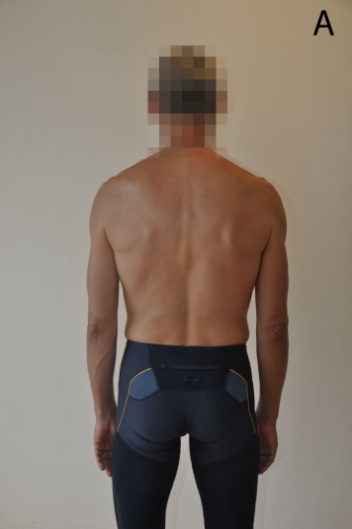
Exercise 2**

**Starting position:** Stand straight in front of a mirror. Arms hanging down by your side (A). Squeeze your shoulder blades gently together and downwards (B).  **Exercise:** Move both arms slightly backwards (C). Maintaining this position rotate your arms slowly inwards and outwards, 3 times (D). Relax.

**Important:** Maintain shoulder blades in correct position throughout the exercise.

**
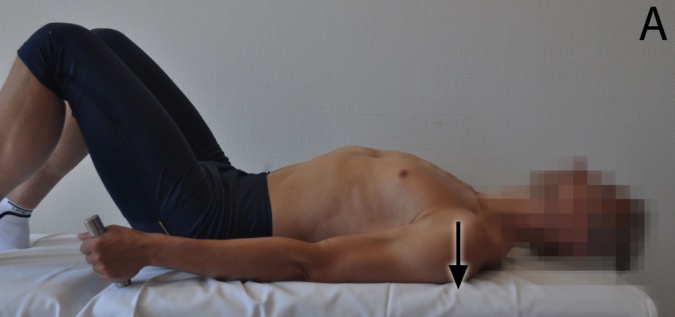

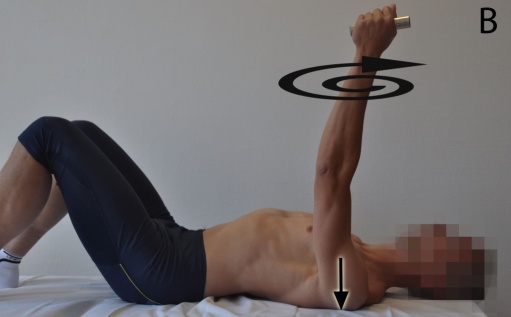
Exercise 3**

**Starting position:** Lie on your back with knees bent, hold a light weight in your hand (A). Raise your arm towards the ceiling being careful not to lift your shoulder blade from the bed/floor.

**Exercise:** Move your arm round in small circles, 3 times in both directions (B).

**Important:** Shoulder blade should remain in contact with the bed/floor during the entire exercise.

**
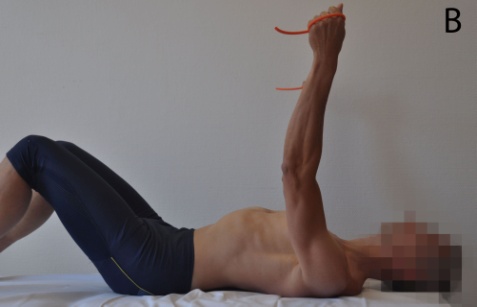

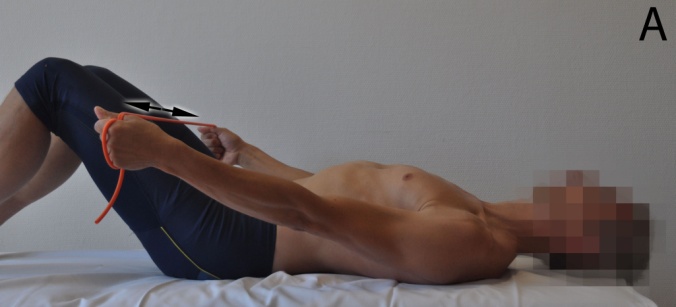
Exercise 4**


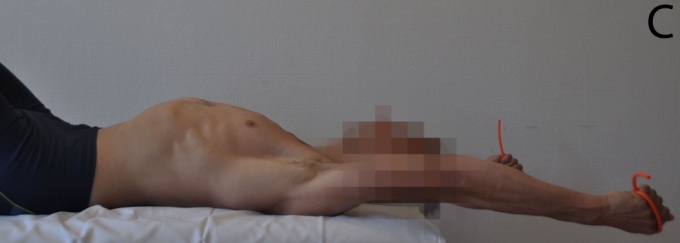


**Starting position:** Lie on your back with knees bent and hold an elastic rope between your hands. Point thumbs up and arms are straight (A).

**Exercise:** Increase the tension in the rope by pulling arms slightly apart, then slowly raise your arms up as far as you can and lower them down again, 3 times (B, C).

**Important:** Maintain contact between shoulder blades and bed/floor during the exercise. Stop the exercise if you feel any pain.
